# Epidemiological and genomic characteristics of *Ralstonia mannitolilytica* isolated from bloodstream in Liaocheng City, Shandong Province, China

**DOI:** 10.1128/spectrum.00812-25

**Published:** 2025-10-07

**Authors:** Fangyuan Cui, Haiying Qian, Xuefeng Miao, Dengying Jia, Shengnan Liang, Lihong Cheng, Hui Yuan, Lijun Huan

**Affiliations:** 1 Liaocheng Center for Disease Control and Prevention 653604, Liaocheng, Shandong, China; 2 Department of Laboratory, Liaocheng Third People’s Hospital, Liaocheng, Shandong, China; 3 Department of Pharmaceutical Inspection, Liaocheng Inspection and Testing Center, Liaocheng, Shandong, China; Universidad Maimonides, Centro de Estudios, Buenos Aires, Argentina

**Keywords:** *Ralstonia mannitolilytica*, cgMLST, multidrug-resistance, comparative analysis, nosocomial outbreaks

## Abstract

*Ralstonia mannitolilytica,* primarily recognized as an environmental bacterium, has emerged as an opportunistic pathogen associated with nosocomial infections. *R. mannitolilytica* is well-adapted to oligotrophic and humid environments, harbors intrinsic resistance to both disinfection measures and antimicrobial therapies and possesses the ability to bypass 0.2 µm filters, thereby facilitating its persistence in clinical and environmental reservoirs. In this study, 11 *R*. *mannitolilytica* isolates were isolated from bloodstream infection cases at a tertiary hospital in Liaocheng City, China, between November 2019 and November 2021. Clinical data, antimicrobial susceptibility profiles, and whole-genome sequencing were analyzed to investigate the resistance mechanisms, genomic characteristics, and clonal dissemination patterns. All isolates exhibited multidrug resistance, exhibiting resistance to ceftazidime, amikacin, gentamicin, imipenem, meropenem, polymyxin B, ceftazidime/avibactam, and chloramphenicol, while retaining susceptibility to minocycline, cefotaxime, cefepime, trimethoprim/sulfamethoxazole, tigecycline, and tetracycline. Core genome multilocus sequence typing and average nucleotide identity analyzes revealed two distinct clades, suggesting independent nosocomial transmission events. Genomic characterization uncovered the presence of intrinsic *bla_OXA-22-like_* and *bla_OXA-60-like_* carbapenemase genes, along with efflux pump genes (*qacJ*, *adeF*), contributing to resistance. Although the infection sources remain unresolved, this study demonstrates the utility of genomic surveillance in tracking hospital-acquired *R. mannitolilytica* outbreaks and advocates for stringent infection control measures to mitigate the spread of this opportunistic pathogen. This report provides novel comparative genomic insights into *R. mannitolilytica*, enhancing understanding of its resistance mechanisms and evolutionary adaptability.

**IMPORTANCE:**

*Ralstonia mannitolilytica*, an environmental bacterium increasingly linked to hospital-acquired infections, poses significant treatment challenges due to its resistance to common antibiotics. This study investigates 11 bloodstream infection cases caused by *R. mannitolilytica* in a Chinese hospital, revealing two distinct transmission clusters through genomic analysis. The findings highlight the bacterium’s ability to persist in clinical settings and its resistance to critical antibiotics like carbapenems, likely driven by intrinsic genes and efflux pumps. By combining patient data and genomic surveillance, this work underscores the urgent need for enhanced infection control measures to curb outbreaks. It also provides novel comparative genomic insights into *R. mannitolilytica*, advancing our understanding of its evolution and resistance mechanisms, which is vital for guiding future clinical management and public health strategies.

## INTRODUCTION

The genus *Ralstonia,* initially described by Yabuuchi in 1995, is a gram-negative, oxidase-positive, nonsporulating rod with a complex systematic history (1, 2). While *Ralstonia* species are typically found in the environment, *Ralstonia insidiosa*, *Ralstonia mannitolilytica,* and *Ralstonia pickettii* have occasionally been identified as pathogens associated with healthcare infections in immunocompromised patients. In 2001, *R. mannitolilytica* was classified as a novel species within the genus *Ralstonia* based on the 16S rRNA gene sequence (3). *R. mannitolilytica* thrives in oligotrophic and moist environments, exhibits resistance against disinfection measures and treatment regimens, and has the ability to penetrate 0.2-µm filters (4–6). Schnetzinger *et al*. analyzed water from infusion heater devices and found that 9.1% of the samples tested positive for *R. mannitolilytica* (7). Numerous cases of *R. mannitolilytica* infection have been described, along with several reported outbreaks in healthcare settings worldwide, resulting from contamination with parenteral fluid, medical equipment (such as oxygen delivery devices and multidose bottles of saline), intravenous catheters, or contaminated disinfectants (6, 8–11). Various hospital-acquired infections caused by *R. mannitolilytica* have been reported, including meningitis, bloodstream infections, spherical pneumonia, endocarditis, osteomyelitis, and peritonitis (4, 12–16). Mohamed *et al.* described an outbreak of *R. mannitolilytica* bacteremia in 16 patients undergoing hemodialysis due to contaminated dialysis water (17). Additionally, clonal transmission of *R. mannitolilytica* has been reported in dialysis patients with bloodstream infections in China (18).

However, there are no clear treatment guidelines or Clinical and Laboratory Standards Institute (CLSI) breakpoints for *Ralstonia spp*. Due to the multi-resistant nature of this genus and the lack of evidence for suitable targeted antibiotic therapies, treating infections caused by *Ralstonia* species is often challenging. Among the different species within the genus, *R. mannitolilytica* appears to be more resistant compared to *R. pickettii* and *R. insidiosa* (19). The treatment of *R. mannitolilytica* infections usually relies on the antibiotic susceptibility profile of the isolates. Data on the mechanism of antibiotic resistance in *R. mannitolilytica* is limited; however, the Class D carbapenemase genes *bla_OXA-22_* and *bla_OXA-60_* in the *Ralstonia* chromosome are commonly associated with intrinsic carbapenem resistance (3).

From November 2019 to November 2021, *R. mannitolilytica* bloodstream infections were identified in 11 patients. The genetics, epidemiological characteristics, and resistance mechanisms were investigated to provide a theoretical reference for the control and treatment of *R. mannitolilytica* bloodstream infections.

## MATERIALS AND METHODS

### Epidemiological investigation and isolation

This case series included clinically significant nonduplicate, culture isolates of *R. mannitolilytica* obtained from suspected bloodstream infections in the microbiological laboratory of Liaocheng Third People’s Hospital in eastern China. These cases occurred between November 2019 and November 2021 in the neurosurgery, intensive care unit (ICU), cardiology, and surgery departments. Clinical and demographic data, including demographics, symptoms, physical examination findings, laboratory results, comorbidities, treatments, and outcomes, were extracted from electronic medical records.

Blood cultures were obtained from peripheral veins, and secondary samples were cultured on blood agar plates (HuanKai Microbial, China) and incubated at 37°C for up to 24 h. The isolates were initially identified by VITEK mass spectrometry (BioMérieux, France). Verified strains were preserved in glycerol at −80°C and subsequently revitalized by subculturing on blood agar plates prior to further analysis.

### Antimicrobial susceptibility tests

All isolated *R. mannitolilytica* strains were tested for susceptibility to various antibiotics using the microbroth dilution method on customized antimicrobial susceptibility testing plates (Sensititre, USA). The following concentration ranges were used for the twofold serial dilution of all agents: minocycline (1–32 μg/mL); polymyxin B (0.25–8 μg/mL); piperacillin/tazobactam (4) (8/4–256/4 µg/mL); cefotaxime (4–128 μg/mL); ceftazidime (4–128 μg/mL); cefepime (4–128 μg/mL); amikacin (8–256 μg/mL); trimethoprim/sulfamethoxazole (1/19–32/608 µg/mL); ampicillin/sulbactam 2:1 ratio (2/1–32/16 µg/mL); gentamicin (2–64 μg/mL); ciprofloxacin (0.5–16 μg/mL); levofloxacin (1–32 μg/mL); ceftazidime/avibactam constant (4) (0.25/4–8/4 µg/mL); chloramphenicol: (4–32 μg/mL); tetracycline (1–16 μg/mL); tigecycline (0.25–8 μg/mL); imipenem (1–32 μg/mL); and meropenem (1–32 μg/mL). All isolates were incubated at 37°C for 20 h under aerobic conditions during antimicrobial susceptibility testing. The minimum inhibitory concentration (MIC) of *Escherichia coli ATCC25922* was determined for quality control. The results were evaluated according to the recommended breakpoints for *Pseudomonas spp*., *Burkholderia cepacia*, and *Acinetobacter spp*. from the CLSI M100 2023, except for tigecycline, for which the results were evaluated using the adapted United States Food and Drug Administration breakpoints. The following MIC values (μg/mL) were used for resistance categorization: minocycline ≥16, polymyxin B ≥4, piperacillin/tazobactam (4) ≥128/4, cefotaxime ≥64, ceftazidime ≥32, cefepime ≥32, amikacin ≥64, trimethoprim/sulfamethoxazole ≥4/76, ampicillin/sulbactam 2:1 ratio ≥32/16, gentamicin ≥16, ciprofloxacin ≥4, levofloxacin ≥8, ceftazidime/avibactam constant (4) ≥16/4, chloramphenicol ≥32, tetracycline ≥16, tigecycline ≥8, imipenem ≥16, and meropenem ≥16.

### Whole-Genome sequencing

Genomic DNA was extracted from the isolates using a QIAamp DNA Mini Kit (Qiagen, Germany). The harvested DNA was detected by agarose gel electrophoresis using a Qubit 4.0 fluorometer (Thermo Fisher Scientific, USA). To prepare the sequencing libraries, the MGIEasy FS DNA Library Prep Set (MGI, China) was used, following the manufacturer’s recommendations. Index codes were added to attribute the sequences to each sample. The whole genome was sequenced using an MGISEQ-200 sequencer with a 2×100 bp paired-end sequencing strategy. As a quality control step, polymerase chain reaction (PCR) adapter reads and low-quality reads from paired ends were filtered out. All good-quality paired reads were assembled using the Microbial Genome Analysis Pipeline (MGI, China). The isolates were further verified by sequence alignment using EzBioCloud (20).

### Molecular epidemiological and comparative genomic analyses

To investigate the epidemiological and genomic characteristics of *R. mannitolilytica*, we analyzed 11 sequenced isolates and 27 assemblies from the NCBI database, the accession numbers for all the isolates are provided in File S2 (found at https://zenodo.org/records/17188885). To determine the phylogenetic relationships, whole-genome DNA-sequence-based average nucleotide index (ANI) analysis of *R. mannitolilytica* was performed using the integrated prokaryotes genome and pan-genome analysis service (IPGA) (21). The assemblies of the 27 *R. mannitolilytica* genomes available in the NCBI database were loaded into Ridom SeqSphere+ software v.9.0.10 (Ridom, Münster, Germany) to create a defined core genome multilocus sequence typing (cgMLST) scheme. Isolates with ≤10 allele differences in the cgMLST target genes were defined as highly related, constituting a clonal cluster (22).

The prokaryotic genomic and comparative genomic analysis pipeline (PGCGAP 1.0.26) was used to perform gene prediction and annotation using Prokka (1.14.6) (23, 24). Phylogenetic reconstruction of the single-copy core protein employed 38 *R. mannitolilytica* genomes and two outgroups (*R. insidiosa* ATCC 49129 and *R. pickettii* K 288). Protein clustering was performed using CD-HIT (4.8.1) (25). Single-copy core gene sequences were subsequently extracted via Perl scripts and aligned with MAFFT (version 7) (26). Concatenated protein alignments were used to infer a maximum likelihood phylogeny in RAx×ML-NG (0.9.0) under the optimal substitution model selected by ModelTest-NG (0.1.7), with branch support assessed through 1,000 rapid bootstrap replicates (27, 28). Additionally, pan-genome analysis was conducted using the Roary software (3.11.2) (29). Protein-coding sequences were aligned against the Comprehensive Antibiotic Resistance Database (CARD; https://card.mcmaster.ca/) using thresholds of 70% identity and 60% coverage. For functional classification of the predicted core genes, BLASTP was used to align the amino acids of the predicted genes against the Clusters of Orthologous Groups (COG) database (30). Perl script “get_flag_relative_abundances_table.pl” and R script “Plot_COG_Abundance.R” were used for comparison and visualization of COG functional categories among different genomes. Analyzes were performed using default parameters for all software unless otherwise specified.

## RESULTS

### Demographic data and clinical outcomes

From November 2019 to November 2021, a total of 11 patients were diagnosed with positive *R. mannitolilytica* bloodstream infections. Table 1 presents the demographic data and the clinical outcomes of the 11 patients. The median age was 67 years (range: 18–76 years), and all patients had underlying diseases, including cerebral infarction (72.7%, *n* = 8), coronary heart disease (9.1%, *n* = 1), open fracture (9.1%, *n* = 1), and cerebral hemorrhage (9.1%, *n* = 1). The majority of *R. mannitolilytica* infections were detected in the neurosurgery department (63.6%, *n* = 7), followed by the ICU (18.2%, *n* = 2), cardiology (9.1%, *n* = 1), and surgery (9.1%, *n* = 1). All strains of *R. mannitolilytica* were isolated from blood, with three strains isolated in 2019, six in 2020, and two in 2021.

**TABLE 1:** Characteristics of cases with *R. mannitolilytica* bloodstream infection (*N* = 11)

Characteristics	No. (%)	*P*-value
Median age, year (range)	67 (18–76)	
Age group (years)		
<49	1 (9.1%)	0.631
50-59	3 (27.3%)	
60-69	3 (27.3%)	
70-79	4 (36.4%)	
Sex		
Females	3 (27.3%)	0.227
Males	8 (72.7%)	
Acquisition ward		
Neurosurgery	7 (63.6%)	0.0105
ICU	2 (18.2%)	
Cardiology	1 (9.1%)	
Surgery	1 (9.1%)	
Presence of underlying disease		
cerebral infarction	8 (72.7%)	<0.001
coronary heart disease	1 (9.1%)	
open fracture	1 (9.1%)	
cerebral hemorrhage	1 (9.1%)	
Specimen		
Blood	11 (100%)	–^*a*^
Date of isolation		
2019	3 (27.3%)	0.307
2020	6 (54.5%)	
2021	2 (18.2%)	
Mortality caused by *R. mannitolilytica*	0 (0%)	–

^*a*^ "–”, not applicable.

### Antimicrobial susceptibility patterns

**Fig 1:**
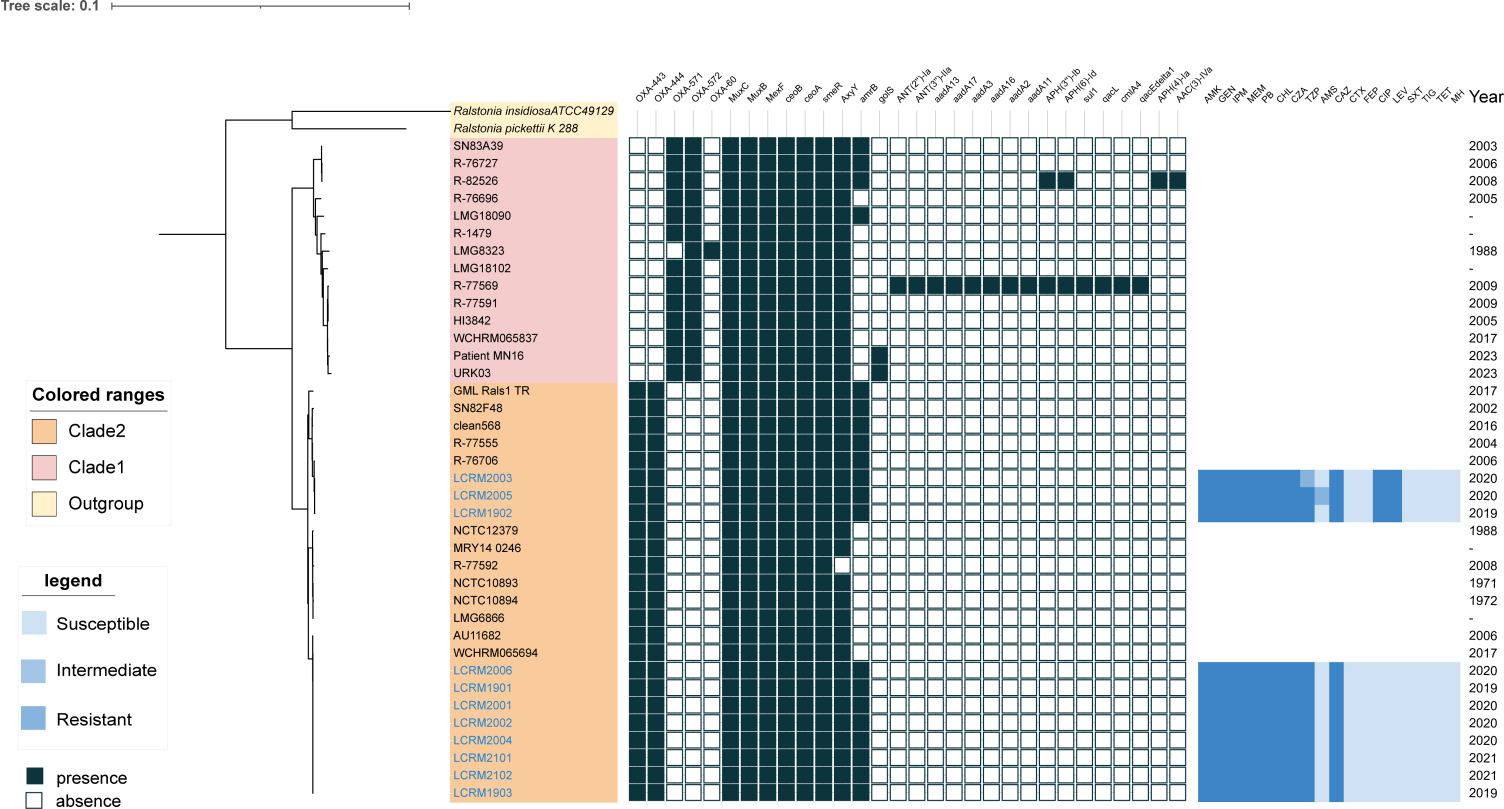
The phylogenetic tree, constructed from single-copy core proteins, presents the antimicrobial resistance genotypes of all 38 *R*. *mannitolilytica* isolates, as well as the phenotypes of the tested subsets. The isolation years are annotated on the right. Dark blue and empty cells indicate the presence or absence of antimicrobial resistance genes, respectively, for each isolate. Color-coded regions differentiate the outgroup and Clades 1 and 2. AMK: Amikacin; GEN: Gentamicin; IPM: Imipenem; MEM: Meropenem; PB: Polymyxin B; CHL: Chloramphenicol; CZA: Ceftazidime/Avibactam Constant 4; TZP: Piperacillin/Tazobactam Constant 4; AMS: Ampicillin/sulbactam; CAZ: Ceftazidime; CTX: Cefotaxime; FEP: Cefepime; CIP: Ciprofloxacin; LEX: Levofloxacin; SXT: Trimethoprim/sulfamethoxazole; TIG: Tigecycline; TET: Tetracycline; MH: Minocycline.

The 11 *R*. *mannitolilytica* strains isolated from the bloodstream in this study were subjected to antimicrobial susceptibility testing; the results are displayed in File S1(found at https://zenodo.org/records/17188885) and Figure 1. The tested isolates exhibited similar antimicrobial susceptibility patterns. All isolates were resistant to ceftazidime (≥32 µg/mL), amikacin (≥256 µg/mL), gentamicin (>64 µg/mL), imipenem (≥16 µg/mL), meropenem (≥32 µg/mL), polymyxin B (≥8 µg/mL), ceftazidime/avibactam (>8/4 µg/mL), and chloramphenicol (≥32 µg/mL) but susceptible to minocycline, cefotaxime, cefepime, trimethoprim/sulfamethoxazole, tigecycline, and tetracycline. LCRM1902, LCRM2003, and LCRM2005 were resistant to quinolones (ciprofloxacin and levofloxacin), whereas the other eight isolates were susceptible. All isolates showed intermediate susceptibility to piperacillin/tazobactam, except for LCRM2003, which showed susceptibility. In addition, 10 strains were susceptible to ampicillin/sulbactam, whereas LCRM2005 showed intermediate susceptibility.

### General genome features of R*. mannitolilytica*

The general information for all 38 genomes, including 11 genomes from this study and 27 obtained from the NCBI database, is summarized in File S2 (found at https://zenodo.org/records/17188885). The 11 *R*. *mannitolilytica* isolates in this study exhibited genome sizes ranging from 4.73 Mb (strain LCRM1901) to 4.93 Mb (LCRM2003), with GC contents of 65.64–65.82%. For the 27 publicly available strains from NCBI, genome sizes varied from 4.51 Mb (R-76706) to 5.38 Mb (HI3842), while GC contents spanned 65.00–66.08%. To determine phylogenetic relationships, whole-genome DNA sequence-based ANI analysis was performed. All tested *R. mannitolilytica* strains demonstrated relatively conserved nucleotide sequences, with ANI values ranging from 95.01 to 99.99% (File S3 [found at https://zenodo.org/records/17188885]), confirming their identification at the species level (31). Relatively high ANI values were observed among the following isolates: LCRM2006/LCRM1901/LCRM2002/LCRM2102/LCRM2101/LCRM2004/LCRM2001/LCRM1903, LCRM2003/LCRM1902/LCRM2005, and NCTC10893/LMG6866/NCTC10894.

### Antimicrobial resistance gene profiles

The main resistance gene profiles obtained from the *in silico* CARD analysis are shown in Fig. 1. Whole-genome sequence analysis revealed that all *R. mannitolilytica* isolates carried novel variant alleles of *bla_OXA-22-like_* and *bla_OXA-60-like_*, except for isolate LMG8323, which carried the known *bla_OXA-60_* gene rather than the novel *bla_OXA-571_*. Interestingly, the isolates in clade one harbored OXA-571/OXA-60-like and OXA-572/OXA-22-like β-lactamases. However, isolates in clade 2 (including 11 strains isolated in this study) possessed OXA-443 and OXA-444, which belong to OXA-22-like β-lactamase and OXA-60-like β-lactamase, respectively. Additionally, *muxB*, *muxC*, *ceoA*, *ceoB*, *smeR*, *axyX*, and *mexF* genes encoding resistance-nodulation-division (RND) efflux pumps were detected in all strains. These efflux systems mediate resistance to multiple antibiotic classes, including aminocoumarins, tetracyclines, monobactams, macrolides, fluoroquinolones, and aminoglycosides. The *clmA4*, *adA3*, *sul1*, *aph(3’’)-lb*, *aph (6)-ld*, *qacL, qacE, aadA11, aadA2, aadA16, aadA17, aadA13, ant (3’’)-lla,* and *ant (2’’)-la* genes were detected in isolate R-77569, whereas R-82526 carried genes encoding aminoglycoside-modifying enzymes, including *aph(3’’)-lb*, *aph (6)-ld*, *aph (4)-la*, and *aac (3)-lVa*.

### The distribution of genes into COGs functional categories

**Fig 2:**
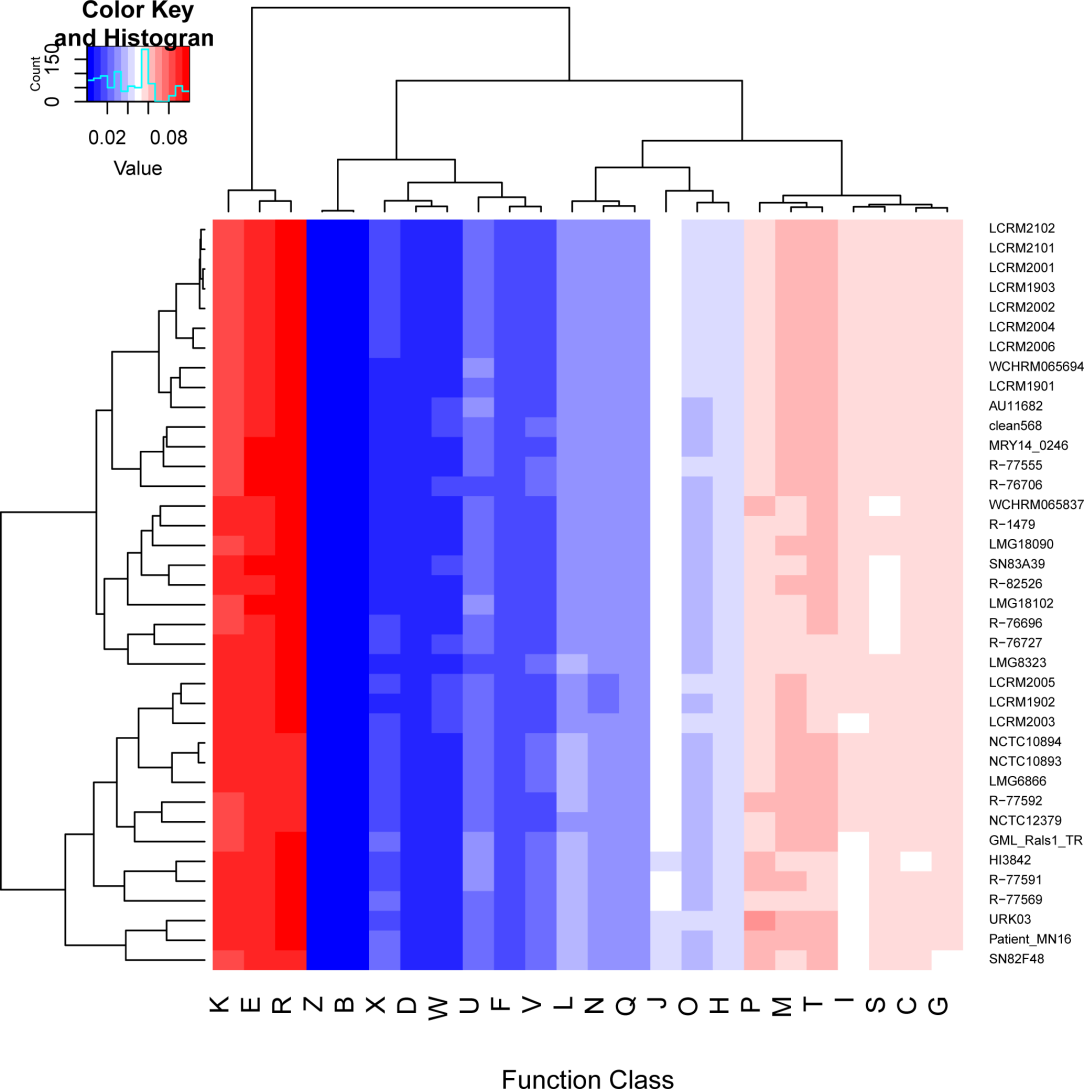
The protein phylogeny of *R. mannitolilytica* is based on the relative abundance of each COG class for all strains. COG classes: K. transcription; E. amino acid transport and metabolism; R. general function prediction only; Z. cytoskeleton; B. chromatin structure and dynamics; X. mobilome: prophages, transposons; D. Cell cycle control, cell division, chromosome partitioning; W. Extracellular structures; U. Intracellular trafficking, secretion, and vesicular transport; F. Nucleotide transport and metabolism; V. Defense mechanisms; L. Replication, recombination, and repair; N. Cell motility; Q. Secondary metabolites biosynthesis, transport, and catabolism; J. Translation, ribosomal structure, and biogenesis; O. Posttranslational modification, protein turnover, chaperones; H. Coenzyme transport and metabolism; P. Inorganic ion transport and metabolism; M. Cell wall/membrane/envelope biogenesis; T. Signal transduction mechanisms; I. Lipid transport and metabolism; S. Function unknown; C. Energy production and conversion; G. Carbohydrate transport and metabolism.

The protein phylogeny of *R. mannitolilytica* genome assembly was determined using the COGs database (Fig. 2). The protein-coding genes of all strains were annotated and categorized into 24 COG terms. At a broad functional level (i.e., COG classes), we observed that the 38 strains had similar functional genome organization. The most dominant COG classes across all strains were ‘amino acid transport and metabolism’ (E), ‘general function prediction’ (R), and ‘transcription’ (K), followed by ‘cell wall/membrane/envelope biogenesis’ (M) and ‘signal transduction mechanisms’ (T). Although the specific distribution of COG classes varied slightly among the individual genomes, COG classes E and R were consistently more prevalent than the other classes in all genomes. Based on the relative abundance of each COG class, the 38 *R*. *mannitolilytica* strains were classified into two clades. Seven sequenced isolates in this study (LCRM2102, LCRM2101, LCRM2001, LCRM1903, LCRM2002, LCRM2004, and LCRM2006) were grouped into clade 1, demonstrating consistent relative abundance and clustering together. LCRM1901 was closer to that of WCHRM065694 obtained from a public database. The other three isolates in our study (LCRM2005, LCRM1902, and LCRM2003) belonged to clade two and tended to cluster in nearby branches.

### Pan-genome analysis of 38 *R. mannitolilytica* strains

**Fig 3:**
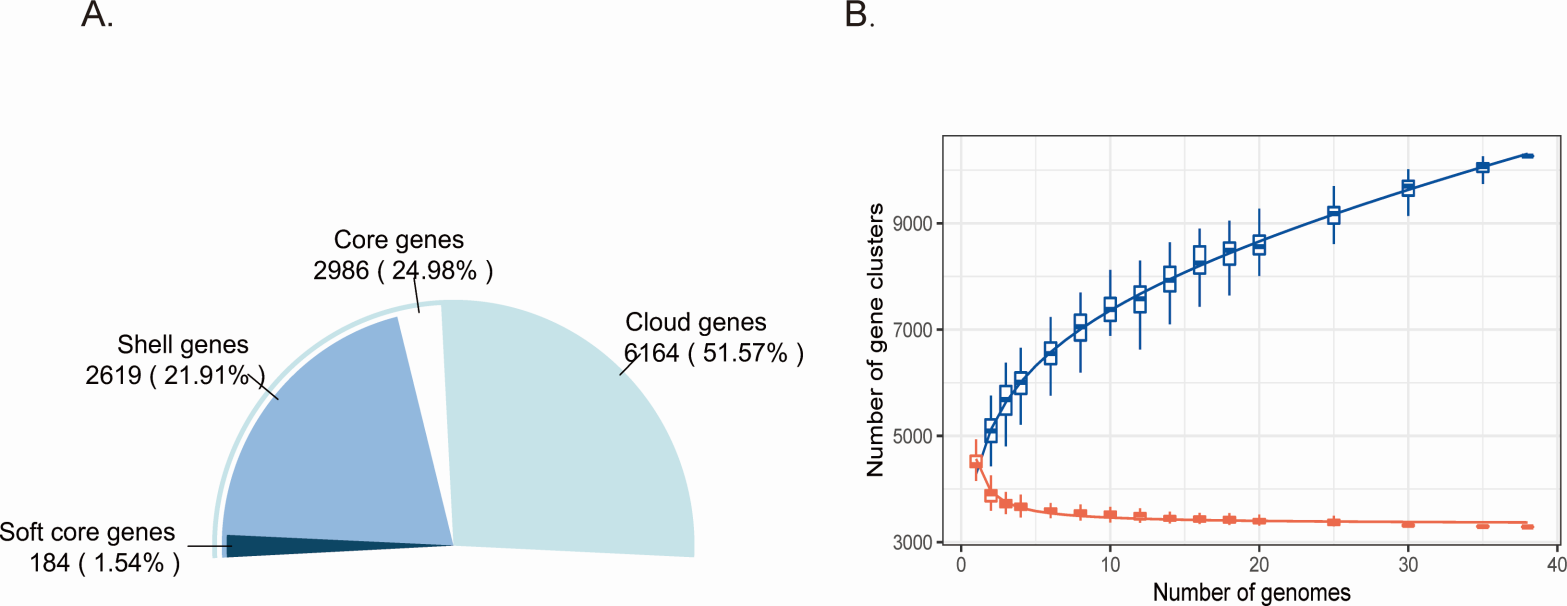
Pan-genome analysis. (**A**) Distribution of pan-genome into core, shell, cloud, and soft core genomes; (**B**) Pan and core genome plot of the 38 *R*. *mannitolilytica* isolates.

In a pan-genome analysis of the 38 *R*. *mannitolilytica* isolates, the genomes were examined, and clusters of orthologous genes were classified into four categories: core, soft core, shell (accessory), and cloud (unique). The pan-genome of the 38 *R*. *mannitolilytica* isolates contained a total of 11,953 coding sequences. Of these, 2,986 (24.98%) were identified as core genes, and the remaining 8,967 constituted the dispensable genome. Notably, cloud genes accounted for 51.57% of the pan-genome (Fig. 3A). The presence of a large pool of dispensable genes from different strains contributed to a relatively large pan-genome size. As shown in the pan-core plot (Fig. 3B), the pan-genome increased as new genomes were added to the analysis, whereas the core genome decreased in size.

### cgMLST and phylogenetic analysis

**Fig 4:**
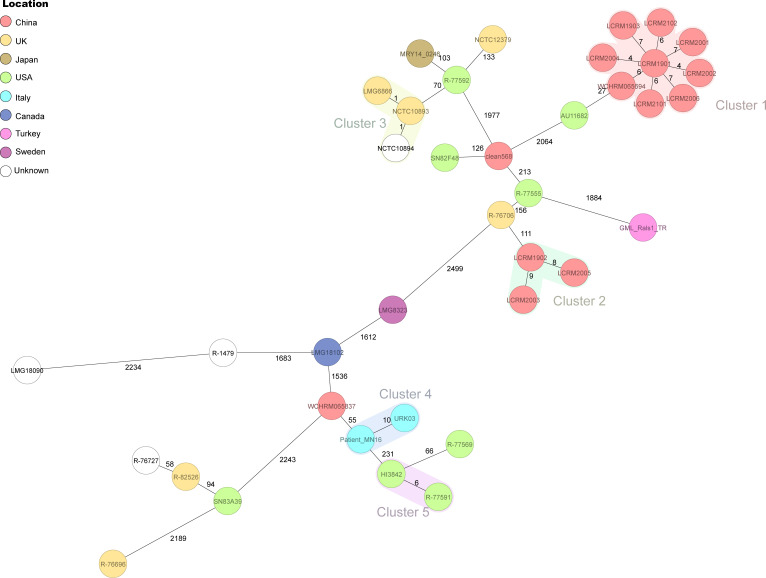
Minimum spanning tree of 38 *R*. *mannitolilytica* isolates showing the genetic relationships based on the cgMLST analysis. Each node represents a single genotype, and the nodes are colored according to the collecting location. Numbers between the nodes indicate the numbers of target genes with different alleles.

A total of 2,933 targets were defined using Ridom SeqSphere+ (9.0.10) to determine the gene-by-gene allelic profiles of the isolates in this study and those derived from NCBI. The results of the minimum spanning tree based on cgMLST are shown in Fig. 4. The tree revealed that most strains exhibited significant allelic differences. Applying a ≤10 allele difference threshold for cluster delineation (22). Based on this threshold, the 11 *R. mannitolilytica* isolates in this study were divided into two distinct transmission clusters. Cluster 1 consisted of 8 strains (including LCRM2006, LCRM1901, LCRM2001, LCRM2002, LCRM2004, LCRM1903, LCRM2102, and LCRM2101) that grouped together with WCHRM065694, which was collected from China in 2017. The other three isolates in Cluster 2 (LCRM2003, LCRM1902, and LCRM2005) exhibited 111 allelic differences from R-76706, which was isolated from the UK in 2006. This indicates that the 11 bloodstream infections in this study represented two different continuous clonal disseminations. Furthermore, several other genetically closely related clusters were identified among the isolates derived from the NCBI database, including cluster 3, cluster 4, and cluster 5.

Figure 1 presents the maximum-likelihood of phylogenetic tree reconstructed from single-copy core proteins. All *R. mannitolilytica* strains cluster into two distinct clades. The 11 clinical isolates from this study are exclusively found within Clade 2. Notably, three isolates (LCRM2003, LCRM1902, and LCRM2005) formed a highly supported subclade, indicating their close phylogenetic relationship. The remaining isolates (LCRM2006, LCRM1901, LCRM2001, LCRM2002, LCRM2004, LCRM1903, LCRM2102, and LCRM2101), together with the reference strains WCHRM065694 and AU11682, formed a sister subclade, showing a conserved genomic divergence.

## DISCUSSION

*R. mannitolilytica* is a gram-negative bacterium that belongs to the *Burkholderiaceae* family and *Ralstonia* genus. It can survive in humid and low-nutrient environments but has now been identified as a rare opportunistic pathogen capable of causing a serious infection in immunocompromised patients (32). The prevalence of *R. mannitolilytica* is increasing owing to the development of modern medical care and inappropriate use of broad-spectrum antibiotics. There have been several hospital outbreaks attributed to *R. mannitolilytica* infection worldwide, although no person-to-person transmission has been reported (10, 11, 17, 18, 32, 33).

In this case series, we described 11 nosocomial cases of bloodstream infections caused by *R. mannitolilytica*, although the source of these infections was not tracked. All patients in this study had underlying serious medical conditions such as cerebral infarction, coronary heart disease, open fracture, and cerebral hemorrhage. *Ralstonia* spp. can cause invasive diseases, but most reports have described a good prognosis and low mortality (33). The most common symptom in these 11 cases was fever, and all patients recovered after therapy. However, owing to the time lag in starting the environmental investigation, the source of the outbreak was not determined. *Ralstonia* outbreaks usually persist for prolonged periods because their sources are usually hypothesized (34). The *R. mannitolilytica* outbreak described in this study lasted for almost 2 years until stringent infection control measures were taken during the COVID-19 pandemic.

Pan-genome analysis was conducted to explore the gene pool, unique genes, and functional information that contribute to bacterial diversification. The number of accessory genes was found to be three times higher than that of core genes, and cloud genes accounted for 51.57% of the genome. These findings demonstrated that *R. mannitolilytica* exhibits significant genomic diversity. The open pan-genome of *R. mannitolilytica* further confirmed its symbiotic relationship with other microorganisms and highlighted its strong capacity for gene flow and adaptive evolution. cgMLST was used to investigate potential nosocomial outbreaks. Based on epidemiological data and cgMLST analysis, the 11 *R*. *mannitolilytica* isolates from this study were divided into two clusters, suggesting that bloodstream infections were independent nosocomial outbreaks. Cluster 1 consisted of eight isolates (LCRM1901, LCRM1903, LCRM2001, LCRM2002, LCRM2004, LCRM2006, LCRM2102, and LCRM2101) that exhibited 4-7 allelic differences. These isolates were responsible for the continuous clonal dissemination between November 2019 and November 2021. Cluster two included three additional strains (LCRM2005, LCRM1902, and LCRM2003) implicated in nosocomial infections during the subsequent period from December 2019 to December 2020.

*R. mannitolilytica* is frequently resistant to many commonly used antibiotics, including several β-lactams and most of the aminoglycosides (32). Siddiqui *et al*. reported 14 cases of nosocomial bloodstream infections caused by *R. mannitolilytica* in India, of which 71.4% (10/14 patients) recovered with the treatment of cefoperazone/sulbactam (10). In our study, all isolates were resistant to aminoglycosides (amikacin and gentamicin), carbapenems (imipenem and meropenem), polymyxin B, chloramphenicol, ceftazidime, and ceftazidime/avibactam Constant 4. Compared with earlier studies in India, where 64.3% of strains were found to be resistant to aminoglycosides and 42.8% showed resistance to carbapenems, our study cohort had significantly higher rates of resistance to both aminoglycosides and carbapenems (10). Susceptibility to tetracyclines (tetracycline and minocycline), cefotaxime, and trimethoprim/sulfamethoxazole was consistent with previous research (32, 33). Most *Ralstonia* spp. are susceptible to quinolones (11, 32). However, the isolates in cluster 2 (LCRM1902, LCRM2003, and LCRM2005) exhibited resistance to quinolones, with high MICs of ciprofloxacin (MIC >16 mg/L) and levofloxacin (MIC = 32 mg/L). Members of the *R. pickettii* lineage, including *R. mannitolilytica*, are inherently resistant to polymyxin (35). Our findings confirmed this, as all 11 isolates showed polymyxin resistance with MIC >8 mg/L. This is an important consideration for clinicians prescribing antibiotics. However, only limited research has been conducted on the resistance mechanisms of *Ralstonia* spp. It has been reported that the class D β-lactamases OXA-22-like and OXA-60-like, which are located on the non-chromosomal replicon, may play an important role in the emergence of carbapenem resistance in *Ralstonia* spp (32, 36). Previous studies have shown that OXA-22 is a narrow-spectrum oxacillinase, and its enzymatic activity primarily targets benzyl penicillin, cloxacillin, and restricted spectrum cephalosporin (37). OXA-66 is an inducible carbapenemase that is capable of hydrolyzing amoxicillin, benzylpenicillin, ticarcillin, and imipenem (38). Our research on resistant genotypes led to the identification of novel variant alleles of OXA-22 in *R. mannitolilytica*, identified as *bla_OXA-443_* and *bla_OXA-572_*, whereas novel variant alleles for OXA-60 were characterized by *bla_OXA-444_* and *bla_OXA-571_*. When studying the distribution patterns of antimicrobial resistance genes among different *R. mannitolilytica* strains, we found that the distribution of *bla_OXA-22-like_* and *bla_OXA-60-like_* variant alleles matched the sequence clustering obtained from the phylogenetic tree of single-copy core proteins. This finding supported previous research and suggested that the *R. mannitolilytica* isolates may represent two subspecies (3). Despite the differences in the resistance phenotypes, no significant variations were detected among the predicted antimicrobial resistance genes at the genomic level in the strains isolated in this study. Multidrug efflux pumps have been consistently identified as key determinants of intrinsic resistance in *Ralstonia spp.* (39). Genomic analysis revealed conserved RND-type efflux pump genes (*muxB/C*, *ceoA/B*, *smeR*, *axyX, and mexF*) across all isolates analyzed in this study, indicating their essential contribution to resistance mechanisms. Specifically, the CeoAB-OpcM system, encoded by the *ceoA/B* genes, demonstrates particular efficacy against fluoroquinolones and aminoglycosides, while the MuxABC-OpmB complex, encoded by the *muxB/C* genes, confers resistance to diverse antibiotic classes, including aminocoumarins, tetracyclines, monobactams, and macrolides (40, 41). Accumulating clinical evidence suggests these efflux systems mediate not only drug extrusion but also virulence regulation and stress adaptation, collectively driving antimicrobial resistance development (42).

Despite these findings, several limitations warrant consideration. First, the limited sample size (*n* = 38) may compromise the reliability of cgMLST-based nosocomial outbreak detection, necessitating increased sample sizes to validate the accuracy of this approach. Second, deeper comparative genomic analyzes, particularly of genetic divergence regions (e.g., resistance gene contexts, accessory elements including plasmids, prophages, and virulence factors) across strains or clusters with distinct resistance profiles were not performed. Finally, the precise source facilitating nosocomial transmission remains unidentified due to the absence of systematic environmental sampling.

### Statistical analysis

Statistical analysis was performed using SPSS 21.0 (SPSS Inc., Chicago, IL, USA). Fisher’s exact test was employed to assess associations between clinical characteristics and *R. mannitolilytica* bloodstream infection. For multinomial distributions (>2 categories), the Freeman-Halton extension of Fisher’s exact test was applied. Results are reported with exact *P*-values, with statistical significance defined at *α* = 0.01 (two-tailed).

### Conclusion

In summary, this study employed cgMLST to identify two independent nosocomial outbreaks of *R. mannitolilytica* bloodstream infections. While the precise sources of transmission remain unidentified, the persistence of cases over a 2-year period underscores the critical need for enhanced infection control protocols in healthcare settings. Our pan-genomic analysis provides the detailed characterization of *R. mannitolilytica*’s genetic diversity and adaptation patterns. The genomic data identify specific resistance mechanisms in this pathogen, enabling more targeted surveillance of hospital outbreaks.

## Data Availability

The data sets generated and/or analyzed during the current study are available in the NCBI database, accession numbers SAMN40150205-SAMN40150215.
